# FOSTERING PRACTICE CHANGE BY INTEGRATING INTERPROFESSIONAL EDUCATION IN A COMMUNITY HEALTH COLLABORATION

**DOI:** 10.1093/geroni/igad104.2393

**Published:** 2023-12-21

**Authors:** Jennifer DeGennaro, Lisa Bodenheimer, Elyse Perweiler, Margaret Avallone, Marilyn Mock, Sreelekha Prakash

**Affiliations:** Rowan-Virtua SOM / NJISA, Stratford, New Jersey, United States; Rowan University, Stratford, New Jersey, United States; Virtual Health College of Medicine & Life Sciences of Rowan University - Rowan-Virtua School of Osteopathic Medicine, Stratford, New Jersey, United States; Rutgers University-Camden School of Nursing, Camden, New Jersey, United States; Fair Share Support Services, Inc., Camden, New Jersey, United States; Stockton University, Galloway, New Jersey, United States

## Abstract

NJGWEP undertook an interprofessional community/public health initiative collaborating with Rutgers University School of Nursing-Camden, Stockton University, NJ Housing and Mortgage Finance Agency, and four affordable housing sites located in underserved communities, with a potential to reach approximately 600 older residents. An IPE curriculum delivered to faculty, students, and staff from multiple health professions disciplines prepares them for assessing residents with chronic health problems using the 4Ms framework (What Matters, Mind, Medications, Mobility), adding the Geriatric 5th M for multi-complexity. Using a Resident Health Risk Assessment (RHRA) to assess residents’ health/biopsychosocial needs by capturing critical information needed to support “aging in place,” students and staff develop person-centered plans of care that address maintenance of independence, health, and referral to needed services/supports to delay or prevent institutional placement. From 2019-2022, 84 students from multiple disciplines (i.e., nursing, social work, counseling, OT) participated in community-based rotations, completed 279 RHRAs, and developed 101 IPE case reviews and person-centered plans of care. Evaluating residents’ co-morbid conditions (Hypertension 46%, Diabetes Mellitus 42%, Mental Health 40%; 8.6 medications per/resident, 44% Mini-Cog < 3; 19% PHQ+, 51% + Falls Screen; 48% TUG>12; 58% ADL/IADL deficits) highlighted the 5th M, Multi-Complexity. IPE is a sustainable venue for training a health care workforce that acknowledges/values the person-centered approach and encourages learning the roles and responsibilities of other disciplines. Implementation of a customized database in 2022 facilitates longitudinal tracking of outcomes related to resident needs, referrals, interventions, access to services to support aging in place, and informs policy and practice change.

